# Flash Sintering Research Perspective: A Bibliometric Analysis

**DOI:** 10.3390/ma15020416

**Published:** 2022-01-06

**Authors:** Eva Gil-González, Luis A. Pérez-Maqueda, Pedro E. Sánchez-Jiménez, Antonio Perejón

**Affiliations:** 1 Instituto de Ciencia de Materiales de Sevilla, Consejo Superior de Investigaciones Científicas−Universidad de Sevilla, Calle Américo Vespucio 49, 41092 Sevilla, Spain; antonio.perejon@icmse.csic.es; 2 Departamento de Ingeniería Química, Universidad de Sevilla, Escuela Politécnica Superior, Calle Virgen de África, 7, 41011 Sevilla, Spain; 3 Departamento de Química Inorgánica, Facultad de Química, Universidad de Sevilla, 41071 Sevilla, Spain

**Keywords:** flash sintering, bibliometric analysis, field assisted sintering, knowledge structure, ceramic materials

## Abstract

Flash Sintering (FS), a relatively new Field-Assisted Sintering Technique (FAST) for ceramic processing, was proposed for the first time in 2010 by Prof. Rishi Raj’s group from the University of Colorado at Boulder. It quickly grabbed the attention of the scientific community and since then, the field has rapidly evolved, constituting a true milestone in materials processing with the number of publications growing year by year. Moreover, nowadays, there is already a scientific community devoted to FS. In this work, a general picture of the scientific landscape of FS is drawn by bibliometric analysis. The target sources, the most relevant documents, hot and trending topics as well as the social networking of FS are unveiled. A separate bibliometric analysis is also provided for Reaction or Reactive Flash Sintering (RFS), where not only the sintering, but also the synthesis is merged into a single step. To the best of our knowledge, this is the first study of this nature carried out in this field of research and it can constitute a useful tool for researchers to be quickly updated with FS as well as to strategize future research and publishing approaches.

## 1. Introduction

Flash Sintering (FS), an electric Field-Assisted Sintering Technique (FAST) [1] for the densification of ceramic materials at a greatly reduced temperature and time, has gained widespread attention since it was established in 2010 by Prof. Rishi Raj’s group from the University of Colorado at Boulder. It basically consists of simultaneously applying heat and a modest electric field to a green body [2] placed on a furnace, allowing the current to totally flow through the sample. The main material requirement is that it should possess a negative temperature coefficient of electrical resistance so that the electrical conductivity increases while heating. At a given applied electric field, there is a critical temperature at which there is a sudden non-lineal rise of the conductivity of the material, which is normally accompanied by instantaneous densification as well as photoluminescence [3]. This signals the flash event and it is now accepted that it is initiated by a thermal runaway induced by Joule Heating [4,5]. Much effort is also being devoted to understanding the underlying mechanisms of FS. A few driven mechanisms have been proposed, but none of them can solely explain the flash phenomenon, which still remains elusive [6,7,8,9,10,11].

Nevertheless, FS has many practical advantages. For instance, it has been proven to be an ecofriendly and versatile methodology, as a wide range of materials from insulators to conductors can be sintered within seconds at furnace temperature much lower than those employed in conventional processing [12,13,14,15,16,17], thereby minimizing the energy footprint. Additionally, in comparison to other FAST techniques such as Spark Plasma Sintering (SPS) [1], it does not require any sophisticated experimental setup; basically just a furnace and a power supply are the two essential components to carry out a FS experiment [18]. An inner working atmosphere is not a prerequisite either, but indeed it is possible to tune it to study its effect over the final properties of the material [14,19,20]. Moreover, some flash-sintered materials have been granted special properties [21,22] and it has been shown that it is possible to sinter unstable oxides with volatile components and complex composition while preserving their stoichiometry and properties [23,24,25,26]. Very recently, in 2018, it was reported that sintering and synthesis can be merged into a single step, giving rise to what has been named Reaction or Reactive Flash Sintering (RFS) and constitutes [27], by itself, another important branch of research.

**Figure 1:**
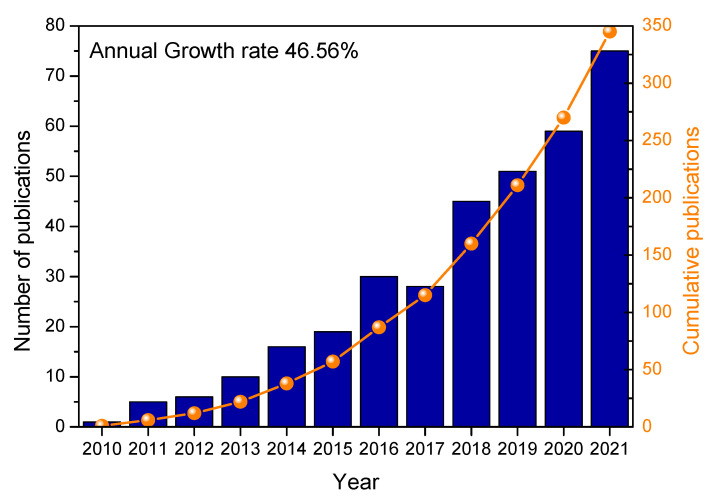
FS annual and cumulative publications from 2010 to December 2021.

Given the advantages of FS, the widespread attention that it has received not only from the scientific community but also from the industry is not surprising [28]. Since it was proposed for the first time a decade ago, this research field has deeply evolved, constituting a true milestone in ceramic processing. The number of peer-reviewed articles has continued dramatically growing year by year with an annual growth rate of about 46% (see Figure 1). To the best of our knowledge, bibliometric analysis [29], which is a powerful tool to quantitatively identify essential variables in a particular research topic (such as top authors, institutions, trends of publication, collaboration, networking, etc.) has never been carried out on FS or RFS. Hence, the aim of this work is to gain insight into the knowledge structure, research perspective, and trends of this fascinating research field. It is noteworthy that the readings of these review articles are highly encouraged [10,18,28,30,31], as a comprehensive literature review is out of the scope of this work. The Biblioshiny web interface of the Bibliometrix R package [32] has been employed to conduct the bibliometric analysis. Peer-reviewed articles and reviews retrieved from the Web of Science (WoS) database have been analyzed based on several bibliometric indicators [33], i.e., quantity (productivity), quality (impact), and structural (networking) indicators. The results derived from this bibliometric analysis allow one to identify the target sources and trend topics in order to strategize future research and publishing approaches. Those publications that most influence this research field have been pointed out. Authors, institutions, and countries in terms of impact and the number of publications have also been identified. Additionally, the social structure of FS is shown by the clustering and collaboration networking from countries to institutions to authors. Moreover, a brief bibliometric analysis is also presented independently for RFS, given its impact and the scientific growth that it has experienced in a very short lifetime (just 3 years).

This article is structured in several sections. The section herein provides a basic and brief introduction about flash sintering, referring to the most relevant literature. The second section explains in detail the methodology used to carry out the bibliometric analysis. The third section provides the most relevant results of this work (target sources, authors, most influential publications, and hot and trending topics) along with data visualization and social networking. The last part of this section is dedicated to RFS. Finally, the conclusions and final remarks are presented.

## 2. Methods

**Scheme 1:**
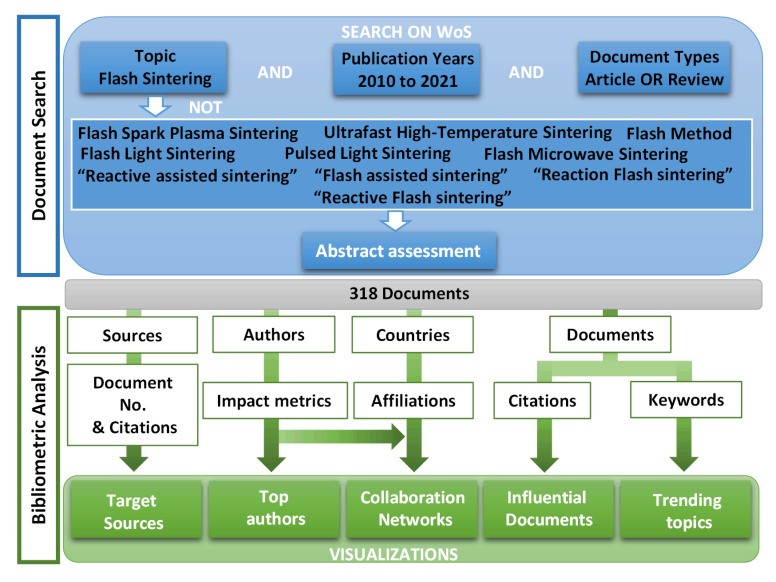
Study design and workflow diagram.

This section presents the design of this bibliometric study, which is schematically depicted in Scheme 1. As explained as follows, it implicitly contains the standard stages of bibliometric analysis, that is to say, the study design, data collection, data analysis, visualization, and interpretation [34,35].

### 2.1. Document Search

Clarivate Analytics Web of Science (WoS), one of the largest databases of peer-reviewed articles in different disciplines, was used for the data collection. The documents included in WoS related to the topic FS were retrieved according to the search strategy shown in Scheme 1, which also includes the specific search query words and Boolean operators. As a search topic, the words Flash Sintering were used, which implies that WoS documents that contain these words in their titles, abstracts, or keywords were scanned. The search was restricted to Flash Sintering, excluding other derived hybrid methodologies such as Flash Spark Plasma Sintering (F-SPS) [36,37], Flash Microwave Sintering [38,39], or Ultrafast High-Temperature Sintering [40], as has been properly indicated in Scheme 1. The reaction or reactive flash sintering documents were also excluded, as a separate bibliometric analysis is provided about this topic (see Table S5). The document types were limited to Articles or Reviews and the time span from 2010 to 15 July 2021, whereas the language was set to English. Finally, manual abstract assessment and screening were performed for each document, resulting in a list of 318 documents, of which the meta-data fields were compiled to conduct the bibliometric analysis.

### 2.2. Bibliometric Analysis

The data analysis was performed with the web interface Biblioshiny of the Bibliometrix R package (Version 1.4). The meta-data files extracted from the 318 documents were converted into R-readable files. Biblioshiny allows an analysis of three metric levels: Sources, authors, and documents. Hence, the most relevant sources, top authors, and documents in terms of productivity and impact can be identified. Additionally, the knowledge structure can be conceptual, intellectual, and socially analyzed, so that thematic mapping and evolution, co-citation, and collaboration networks can be built, among other things.

The metrics analyzed and the derived results from this analysis are schematically depicted in the workflow diagram in Scheme 1. The source analysis points out the target sources, which are the journals where most of the documents are published. The author analysis as well as their countries and affiliations allow one to identify not only the most prolific authors but also the social structure of the FS community, showing co-authorship, institutional, and international networking. The document-level study identifies the most important publications in the field, while the keyword analysis provides a general overview of the most-studied topics and evolution over the years, which makes it possible to identify mainstream and trend topics.

## 3. Results and Discussion

### 3.1. Flash Sintering (FS)

#### 3.1.1. General Descriptive Information

Table 1 summarizes the essential information extracted from the analysis of the set of 318 documents retrieved from WoS from 2010 to 2021, using the word search query indicated in Scheme 1. Detailed information about each metric level is commented as follows.

#### 3.1.2. Scientific Production

FS has constituted a breakthrough in material processing, and since its inception in 2010, it has become a hot topic and a new paradigm in ceramic processing. As it is shown in Figure 1, the number of publications has kept on growing, with an average of approximately 32 documents per year and an annual growth rate of 46.56%. Note that hybrid methodologies such as F-SPS and Reaction or Reactive FS (RFS) have been excluded and only peer-reviewed articles and reviews have been considered. Indeed, the cumulative publications since 2010 follow an exponential trend, as Figure 1 shows. If the topic development continues along the same trend, by a simple fitting to an exponential growth equation, the predicted number of publications in 10 years will be 4400, one order of magnitude higher than nowadays.

There are probably several reasons behind such dramatic growth in such a short period of its lifetime. The main reason is, of course, the scientific relevance of FS for the scientific community, mainly for those interested in ceramic research. Moreover, the simplicity of the FS experimental setup is significant, and as mentioned, just a furnace and a power supply are strictly needed, unlike other FAST methodologies that require complex and expensive equipment [1]. Despite its simplicity, FS is an extremely powerful sintering method that can be successfully used for most ceramic materials, from dielectrics (BaTiO_3_ [41,42,43,44,45,46,47] or (Bi_0.2_Na_0.2_K_0.2_Ba_0.2_Ca_0.2_)TiO_3_ [48]) to ionic (Zirconia, YSZ [2,49,50,51,52], CeO_2_ or doped-CeO_2_ [53,54,55,56,57,58]) or electronic (TiO_2_ [19,22,59,60,61,62], BiFeO_3_ or substituted-BiFeO_3_ [24,27]) conductors. Interestingly, it can be also applied for processing ceramic composites of complex stoichiometry, metastable phases, or materials constituted by volatile species at the temperatures required for their sintering such as YSZ-Al_2_O_3_ composites [63,64,65], different types of solid state electrolytes [25,66,67], BiFeO_3_ [68,69], or K_0.5_Na_0.5_NbO_3_ [26,70,71,72,73]. Moreover, ceramics prepared by FS present very interesting properties rarely reported for materials obtained by convectional procedures. For example, it has been observed that FS specimens deform plastically before fracture when compressed at high strain, due to their extraordinarily high density of defects, such as stacking faults, dislocations, and twins [22,74] or that chemically inert ceramics are converted into active catalytic compounds by enhancing the concentration and reactivity of the ionic species [21,75]. Furthermore, the understanding of the flash phenomena still remains elusive and requires contributions from different scientific fields. This challenge has raised the interest of many researchers with different expertise, including theoreticians and experimentalists [76,77,78,79]. Another significant feature of FS is its ecofriendly nature and potential to possibly scale-up, as it requires less energy than conventional processing processes [80] and, therefore, it contributes to reducing CO_2_ emissions. The possibility of working under continuous FS with rolling electrodes has been proposed in the literature [81]. This idea is already being explored at a larger scale by the company Lucideon [82] in a pilot plant. Very recently, the possibility of homogenously sintering 3D-complex shaped ceramics by the application of a three-phase power supply has been reported, giving rise to what has been named Multiphase Flash Sintering (MPFS) [83]. MPFS is presented as a feasible option to overcome the shape restrictions of conventional FS specimens. Moreover, the capabilities of FS combined with other FAST techniques are evolving in other interesting “flash-based methodologies” that have experienced great development as well. That is the case of Flash Spark Plasma Sintering (F-SPS), where pressure and pulsed currents are simultaneously applied, enabling the homogeneous and energetically efficient sintering (by different electrodes architectures) of both electric conductive and insulating materials [36,37,84,85,86,87,88]. Contactless Flash Sintering (Contactless-FS) is another flash-based methodology. Plasma electrodes are used instead of the traditional metallic wires. The plasma not only heats the material but also carries the current to trigger the flash, minimizing some of the thermal management issues encountered in conventional flash sintering due to the sample–electrodes contact [76]. Similarly, Flame-assisted Flash Sintering (FAFS) uses a flame as an electrode and heating source [89]. It has proved to be an effective technique for the sintering of ceramic coatings on metallic substrates. The combination of FS with Cold Sintering has resulted in Cold Flash Sintering (CFS), where the presence of relatively small amounts of liquids, such as water of acetic acid, on the pellets are used as electrolytes and enables the flash event even at room temperature [90,91]. Last but not least, the efforts made by the pioneers in the field, Prof. Raj and others, have been relevant by spreading the topic through the scientific community by inviting visitors to their labs, collaborating with other groups, giving lectures, and organizing successful International Conferences on the topic “Electromagnetic/Electric Fields in materials processing” such as those held on 2016 and 2019 in Tomar (Portugal) or the symposiums arranged by the Materials Research Society (MRS).

#### 3.1.3. Source-Level Analysis

**Figure 2:**
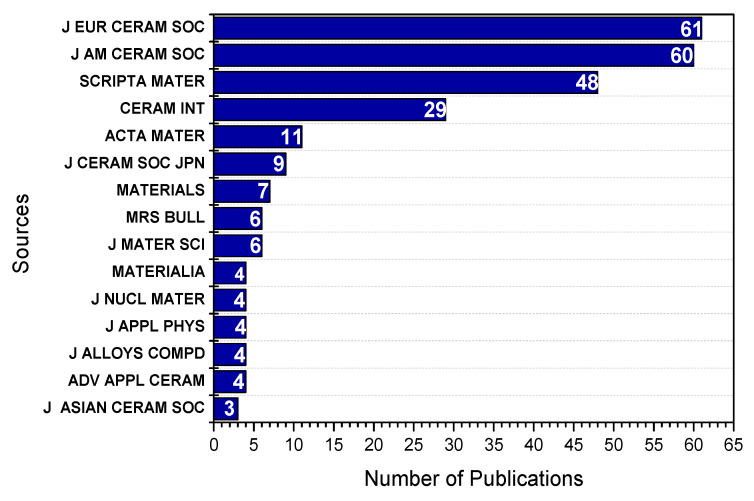
Top 15 sources with 3 or more published papers.

As mentioned, just peer-reviewed articles have been considered in this literature set. The analysis reveals that the documents have been published in 63 different journals (Table 1). Nevertheless, a close examination of Figure 2, which includes the sources where most of the articles have been published, shows that FS documents are concentrated in a few journals, i.e., *Journal of European Ceramic Society* and *Journal of the American Ceramic Society*. Indeed, those two are the core journals where more than one-third of the entire collection has been published. Additionally, these two journals together with *Scripta Materialia (Letters journal of Acta Materialia)*, *Ceramics International*, and *Acta Materialia* have published more than 65% of the analyzed set of documents. These journals are mainly targeted towards ceramic materials as well as their relationship between processing, microstructure, and properties, which makes sense as the vast majority of flash-sintered materials are ceramics provided that a negative temperature coefficient of electrical resistance is possessed [28]. It is noteworthy that these five journals are top-ranked journals of the first quartile according to the Journal Citation Report (2020) in the category of Material Science–Ceramics (*Journal of European Ceramic Society*, *Ceramics International*, and *Journal of the American Ceramic Society*) or Metallurgy and Metallurgical Engineering-Science (*Scripta* and *Acta Materialia*). Reciprocally, these journals are also the most locally cited sources in the set of documents analyzed (see Table S1). A locally cited source is a journal included in at least one of the reference lists of the analyzed document collection. Additionally, there are also articles devoted to FS in general high-impact journals such as *Nature Communications* [74] or *Science Advances* [22].

This simple source-level analysis shows that FS research is mainly published in high-ranked, peer-reviewed journals and provides an idea about the high quality of the research carried out in this field and the interest that it generates and attracts within the scientific community.

#### 3.1.4. Author-Level Analysis and Networking

As shown in Table 1, the author-level analysis reveals that this set of documents involved 670 authors from 29 different countries. Seven of those authors published single-authored documents, whereas the rest participated in co-authored documents. Generally speaking, each document is written by 2.11 authors on average and the Collaboration Index, defined as the total authors of multi-authored documents divided by the total number of multi-authored articles, is 2.2. Additionally, Figure S1 (Supplementary Materials) depicts the country’s scientific production map, where it is qualitatively shown that the USA, China, and Italy are the most involved countries.

Table 2 includes a list of authors that have published, so far, more than 10 articles directly related to FS along with their local *h*-index. The local *h*-index has been calculated using the conventional procedure (number *h* of publications that have been cited *h* times or more [92]) but only considering the set of 318 documents analyzed here. More details about authors with the highest numbers of local citations (citations included in this set of 318 documents) and global citations can be found in Table S2. It must be noted that those lists have been exclusively elaborated with the information retrieved from articles directly related to FS, as explained in the methods section. Those dealing with other similar techniques, such as F-SPS and others, have not been contemplated in the lists. In any case, Table 2 and Table S2 should not be considered as any type of author’s ranking, as most authors in those lists have a significantly larger number of publications and citations in different related scientific topics that have not been included here. Moreover, some authors have been involved in FS almost since the early days, while others have become involved in recent times. As a way of example, the production of the authors included in Table 2 over the 11-year period of lifetime of FS is depicted in Figure S2, where the bubble size is proportional to the number of documents and the color darkness to the citations. Regardless of this, Prof. Raj not only introduced the FS topic for the first time in 2010, but he is the most prolific author in terms of the quantity of papers, citations, and the local *h*-index.

**Figure 3:**
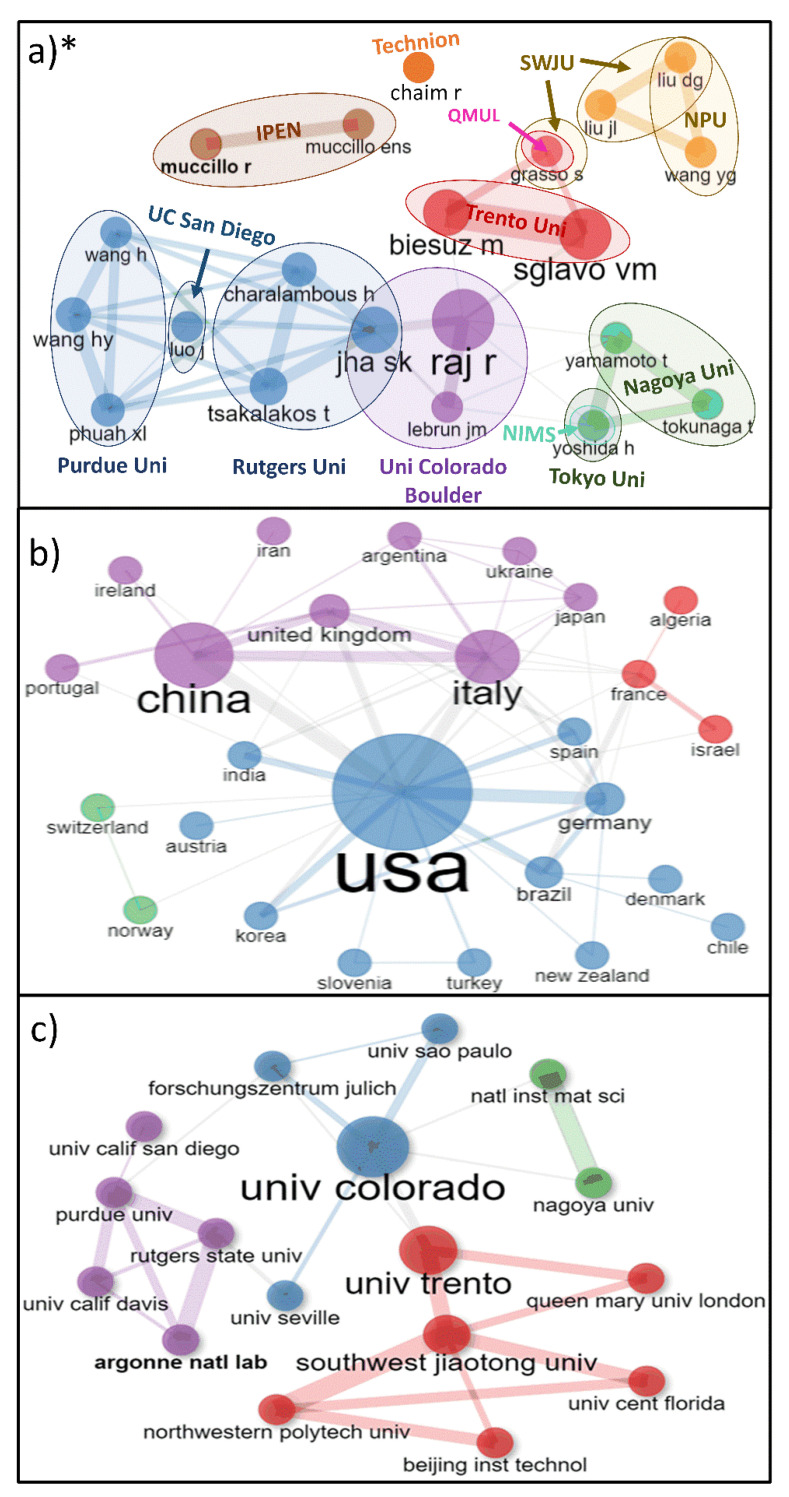
(**a**) Selected authors from Table 2, (**b**) countries, and (**c**) institution’s collaboration networks. * UC San Diego: University of California San Diego, IPEN: Instituto de Pesquisas Energéticas e Nucleares, QMUL: Queen Mary University of London, SWJU: Southwest Jiaotong University, NPU: Northwestern Polytechnical University, NIMS: National Institute for Materials Science, Technion: Technion–Israel Institute of Technology.

The high level of interaction among authors working in FS is very relevant, as shown in the collaboration maps of Figure 3. This type of figure displays the knowledge structure of the research field by providing a general overview about its social structure or, in other words, how the FS scientific community interacts at different levels (authors, institutions, and countries) [35]. Qualitatively, the bubble size in these collaboration maps represents the number of documents, while the strength of the relationship is represented by the links; the thicker, the stronger. Moreover, the position represents the influence, placing the most influential items, i.e., author, institution, or country, at the center of the maps. The different colors or clusters denote common collaboration networks or sub-networks. Figure 3a includes the collaboration map just among authors included in Table 2. Interestingly, all authors in Table 2, apart from Prof. Chaim, have very strong collaborations with others from the same list. Actually, in some cases, they even belonged to the same research group. That is the case of Biesuz who was a former PhD student in Sglavo’s lab (Trento University, Trento, Italy), Charalambous in Tsakalakos’ lab (Rutgers University, New Brunswick, NJ, USA), H Wang and Phuah in Haiyan Wang’s lab (Purdue University, West Lafayette, IN, USA), while Lebrun was a postdoc in Raj’s lab (University of Colorado at Boulder, Boulder, CO, USA). Moreover, there is a significant movement of authors from one institution into another that is helping to spread the topic. For instance, Jha who is currently an Assistant Professor at the Indian Institute of Technology Kanpur was a former PhD student of Raj and, later on, a postdoc in Tsakalakos’s lab. Dianguang Liuwas a former PhD student at Northwestern Polytechnical University (working with Wang, Yiguang) and then moved to Southwest Jiaotong University where he works with Jinling Liu. Grasso is currently a professor at the Southwest Jiaotong University after having worked at the Queen Mary University of London. Moreover, as seen in Figure 3a by the solid connectors, there are many fruitful collaborations among different groups, resulting in co-authored publications. As mentioned, the behavior observed in Figure 3a is limited to authors from Table 2. Nevertheless, it is quite general and can be extrapolated to the whole FS community, as explained as follows by Figure 3b,c, which represents the countries and institutional collaboration maps, respectively, and aims to provide a broader overview of the scientific landscape of FS. As depicted in Figure 3b, there are authors from 26 different countries with at least one mutual publication. Note that the whole FS community involved authors from 29 countries, as is shown in Figure S1. This highlights, once again, the strong interconnection of this scientific community. Similar to Figure 3a, the USA is the country with the highest number of publications and the strongest collaboration network. Authors from China and Italy also have important scientific production and collaboration networks. Interestingly, even authors from countries such as Germany, Spain, India, and France, among others, have become involved in FS more recently and, therefore, while their number of publications is not that high, they have very strong collaboration networks. For example, in the whole document set, only five papers have been issued by corresponding authors from Spain, all of them being internationally collaborative publications. Figure 3c shows the collaboration network at an institutional level. The FS community involves authors from 217 institutions. Thus, for the sake of clarity and visualization, the institutional collaboration map has been limited to those 18 institutions with the highest number of published papers. Analogously to Figure 3a, the bubble size of the University of Colorado at Boulder (USA) and Trento University (Italy) is not surprising; as mentioned, the most-productive authors in terms of publications belong to these institutions, i.e., Raj, Sglavo, and Biesuz. Moreover, both institutions have strong collaboration networks worldwide. For instance, Trento University, Southwest Jiaotong University, one of the most prolific Chinese institutions, and Queen Mary University of London (UK) have established a strong collaboration sub-network. As commented for Figure 3a, Southwest Jiaotong University and the Queen Mary University of London are the current and previous affiliations of Prof. Grasso, respectively. At the same time, other researchers from Southwest Jiaotong University (Liu Dg and Liu Jl among others) work with researchers from other academic centers, such as the Northwestern Polytechnical University (China) and the University of Central Florida (USA). Additionally, Figure 3c also unveils a strong collaboration sub-network within American institutions composed of Rutgers University, Purdue University, Argonne National Laboratory, the University of California San Diego, and the University of California Davis. In albeit extreme simplification, this subnetwork is partially shown in Figure 3a, proving again its similarities with the whole FS scientific community. Most of the works of this American co-authorship sub-network involve some kind of in situ measurements during FS or to flash-sintered samples, such as energy-dispersive X-ray diffraction [15,53,69,93].

All in all, Figure 3 highlights the significant level of interaction among research scientists and groups in FS. It is evidenced that authors involved in this research field are quite eager to collaborate with others from different institutions and even countries. Thus, internationalization is at the core of FS. This practice may be related to the complexity of the FS process that involves physical and chemical phenomena. Thus, its understanding and implementation demand an interdisciplinary approach from Physics to Chemistry and Engineering, and it requires of the participation of both experimentalists and theoreticians. Moreover, collaborations with experts from large scientific facilities (National Laboratories) in the monitoring of the FS process under in situ conditions are also quite noticeable. This strong collaborative networking as well as the International Conferences in Tomar (Portugal) and MRS symposiums, mentioned above, are helping to create a sense of community among researchers worldwide, and it is probably contributing to the fast development of this relatively new research field.

#### 3.1.5. Document-Level Analysis. Influential Documents and Trending Topics

This section studies the most influential documents of the collection with respect to their number of citations as well as the most commonly tackled topics in FS by keyword analysis.

Table S3 includes the top 20 most-cited documents, where the number of local and global citations as well as their ratio are shown. A local citation refers to the number of citations that one document has received from the analyzed document set (in this case, 318 documents and all of them related to FS), whereas global citations are the total number of citations from WoS that may include documents that are not necessarily associated with the analyzed research topic. Therefore, in principle, the higher local citations, the more important and influential the document is in a particular research field. This list is ranked by the paper of Cologna, Rashkova, and Raj reporting the first demonstration of FS, which triggered the development of this research field [2]. This paper constitutes a true milestone in ceramic processing and to date has received more than 474 citations, 270 of which correspond to local citations. This is followed by a single-authored paper of Raj, addressing the role of Joule Heating during FS [8]. It entails one of the first works devoted to explaining the underlying mechanisms of FS, concluding that the fast sintering rates achieved in FS cannot be solely explained by Joule Heating. This work closely links with another top-ranked paper (fourth place) authored by Todd et al. [4]. The authors modelled the electrical and thermal response of 3YSZ under FS conditions and established that the flash event is triggered by a thermal runaway caused by Joule Heating. Indeed, it is now well-accepted within the scientific community that the thermal runaway induced by Joule Heating is the actual phenomenon that initiates the flash. The third most-cited paper both globally and locally corresponds to the work of Cologna et al. published in 2011 [13], which deals with the demonstration of FS in alumina, a highly insulating material. Finally, special attention is deserved for the paper ranked fifth, as this is the second literature review dedicated integrally to FS [28] (to the best of our knowledge, the first review was published by Dancer in 2016 [30]). It was published in *Advances in Applied Ceramics* in 2017 and, to date, has received more than 207 citations, where half of them correspond to local citations. It is also worth mentioning that 75% of the top 20 most-cited documents in FS are published in either the *Journal of the European Ceramic Society* or the *Journal of the American Ceramic Society.* As mentioned in the source-level analysis section, both are high-ranked journals dedicated to the study of ceramic materials, which once again highlights the quality of the research carried out by the scientific community of this field.

In order to provide some perspective about influential works recently published, we carried out a similar analysis while refining the time span to 2020 and 2021. Thus, Table S4 presents the most global and locally cited documents in 2020 and 2021. Most of these publications deal with the understanding of the FS mechanisms in ceria and titania [62,94] or the correlation between the effect of the experimental parameters and the induced defects on the final properties and microstructure of the flash-sintered materials [51,95,96,97,98]. Many of these documents are co-authored by early-stage researchers, such as Lavagnini [51] and Storion [96], both PhD students at the University of São Paulo (Brazil), or Phuah [95,98] and Mishra [94,97] who recently obtained their PhD degrees from Purdue University (USA) and Forschungszentrum Jülich (Germany), respectively. This is an indication that many early-stage researchers are developing their careers in FS and are making important contributions to the field.

Keyword analysis is commonly used in bibliometric analysis to systematically identify the document content, trend topics, and research hotspots of a particular research field. Table 3 contains the top 10 Keywords Plus and Authors’ Keywords. Authors’ keywords are provided by the authors themselves, whereas Keywords Plus are generated by an algorithm, extracting words that frequently appear in the title’s references and not necessarily in the title of the articles or as Author Keywords [99]. Very recently, a study carried out by Zhang J. et al. reveals that both types of keywords identify very similar research trends and knowledge structures [100]. Indeed, the list of words included in Table 3 for both categories as well as the prevalence order is quite similar. Note that trivial keywords, such as Flash Sintering or Flash Sintered, have been cleaned. An examination of these keywords reveals that zirconia is the most-studied material. As mentioned, the first demonstration of FS was carried out with this material [2]. Since then, zirconia powders of different compositions have been widely used as a model material to study the underlying mechanisms of FS as well as the driving sources triggering the flash event, which is also linked to other top keywords such as Joule Heating, thermal runaway or defects. As a way of example, a few works are cited herein [4,50,101,102]. Another important part of the research carried out in FS deals with the study of the properties of the flash-sintered materials, such as their microstructure, abnormal grain growth, defect structures, etc. [22,103,104], some of them granted with special properties. This also explains some of the top keywords obtained, e.g., “microstructure”, “grain growth”, “defect structures”.

**Figure 4:**
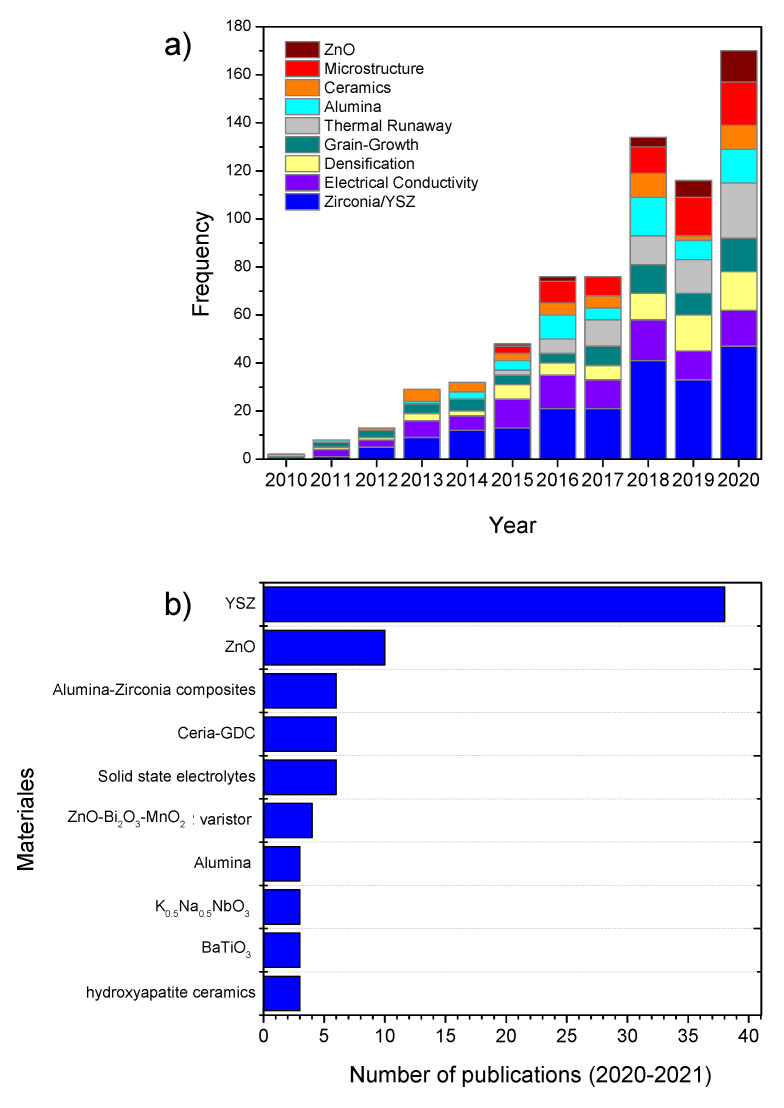
(**a**) Keywords Plus dynamic and (**b**) top 10 studied materials during the two-year period 2020–2011.

Figure 4a shows the Keywords Plus dynamic, representing the frequency of each keyword as a function of time (from 2010 to 2020), which allows for identifying trend topics and research hotspots. For instance, it can be observed that zirconia has always been a hot topic in FS. As mentioned above, it is now well accepted that the flash event is triggered by a thermal runaway. Since it was proposed in 2015 by Todd et al. in the already-mentioned paper entitled “Electrical characteristics of flash sintering: thermal runaway of Joule heating” [4], the keyword thermal runaway has continued growing. Therefore, the contribution of Todd R.I. et al. constitutes an important milestone in FS. From Figure 4a, is also depicted that ZnO is currently a trending topic, and in the last few years its annual occurrence has been increasing. In a similar way to zirconia, ZnO has been widely employed as a model material in FS [103,105,106]. This Keyword Plus dynamic analysis agrees well with Figure 4b, which includes the top 10 most-studied materials during 2020 and 2021. As expected, Zirconia leads the ranking, with more than 38 publications, followed by ZnO. Figure 4b also reveals that another important body of work in FS is dedicated to the sintering of materials with technological interest, which development is being hampered by the high temperatures required or other kinds of difficulties in their processing such as the volatilization of some of their components. That is the case of different sodium and lithium ion conductive ceramics for solid-state batteries [67,107], the lead-free piezoelectric ceramic potassium sodium niobate [70,73], or ZnO-Bi_2_O_3_-based varistor ceramics [108,109,110]. We expect that this trend will probably be maintained during the next few years. That is to say that Zirconia and ZnO will continue being hot topics. As mentioned, the FS mechanisms are still clouded, and further studies about their understanding will certainly be carried out. Therefore, it is quite likely that these two materials will continue to be used as models in future works dealing with the underlying nature of FS. On the other hand, besides the reduced temperatures and times offered by the FS technique, it has also proved to be an engineering tool to grant special and unexpected properties to materials [22,75]. Thus, researchers will continue exploring these FS capabilities and, therefore, the study of the properties of the materials prepared by FS as well as the preparation of new ceramic materials that are hard or impossible to prepare by conventional procedures will be another mainstream area of research.

All in all, we would like to emphasize that this keyword analysis provides a general and brief overview of the document content as well as of the major tackled topics and future research prospects in FS. It is probably quite trivial for researchers who have been working in the field for a while but may be useful to strategize future research as well as a starting point for those researchers who are not acquainted but keen on FS.

### 3.2. Reactive Flash-Sintering (RFS)

**Figure 5:**
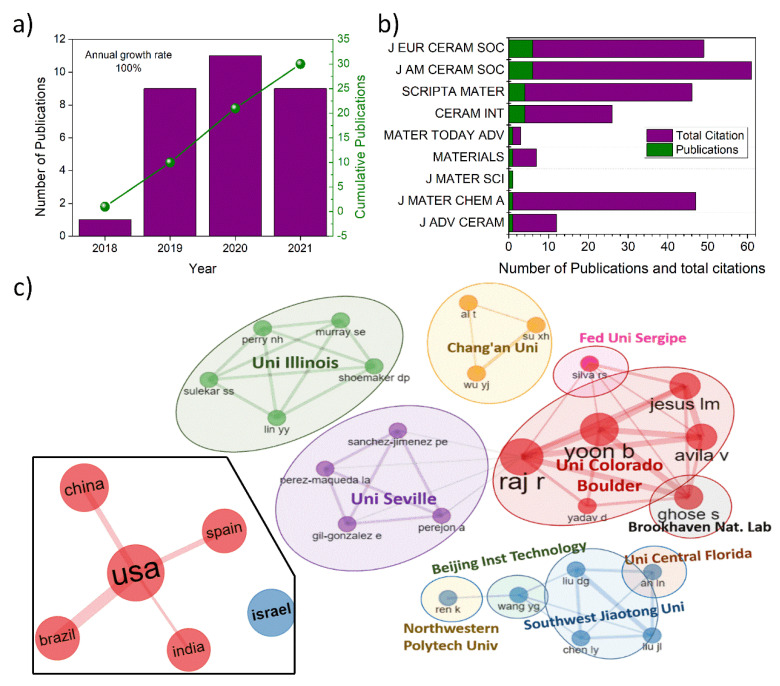
(**a**) Annual and cumulative publications, (**b**) number of documents and total citations per source, and (**c**) country, author, and institution collaboration networks in RFS. The author and institution collaboration networks are limited to those with more than two publications and at least one co-authored document.

As mentioned in the introduction, another important branch of research directly related to FS is Reaction or Reactive Flash sintering (RFS). The foundations and the working mode are quite similar but instead synthesis and sintering are merged in a single step. RFS was reported for the first time in 2018, showing that a highly dense and pure metastable oxide can be prepared in a matter of seconds from a mixture of its basic constituents [27]. RFS soon garnered the attention of the scientific community and, since then, many documents have been published exploiting the capabilities of RFS. Indeed, Figure 5a shows the scientific production for RFS with a remarkable number of 32 publications in just three years, which implies a dramatic annual scientific production growth rate of 100%. Thus, due to the relevance of RFS, we decided to carry out a separate bibliometric analysis to sketch the general scientific map for RFS. The specific word search query as well as the general descriptive information from the analyzed document set for RFS can be found in Tables S5 and S6, respectively.

As can be observed from Figure 5b, RFS follows the same trend as FS in terms of targeted sources. *The Journal of the American Ceramic Society* and *the Journal of the European Ceramic Society* are where almost 50% of the entire collection have been published. Those two journals together with *Ceramics International* and *Scripta Materialia* published 80% of the entire collection. All of them are highly ranked journals according to Journal Citation Reports (2020) and accumulate most of the citations, which highlights, once again, the quality and relevance of the topic.

Nevertheless, the most-relevant publication of this set of documents is probably from *the Journal of Materials Chemistry A*. This journal published just one paper about RFS, but, by itself, it accumulates 20% of the global citations of the entire set. This document was authored by Gil-Gonzalez et al. in 2018 [27] and constitutes, to the best of our knowledge, the first demonstration of RFS. It has received 46 global citations with an average of 11.50 citations per year. Additionally, it is also the most-cited document of the set. Seventeen documents out of twenty-five have cited it, which makes this paper the most relevant one for the research topic of RFS. Details about the historical direct citation network in RFS can be found in Figure S3. Further details about authors, document impact, in terms of quality and quantity, as well as the most studied materials in RFS can be found in SI (Tables S7–S10). It is worth mentioning that according to Table S10, RFS has been mostly dedicated to the preparation of materials with technological interest. This is probably due to the advantages of reduced temperatures and times offered by the technique. A significant number of works have been dedicated to the preparation of high-entropy oxides [111,112,113,114,115], followed by ceramics such as the multiferroic BiFeO_3_ and related materials [27,80,116], solid electrolytes [117,118,119], or the lead-free piezoelectric potassium sodium niobate [120,121]. Analogously to Zirconia or ZnO in FS, the underlying mechanisms in RFS have been studied by high-resolution in situ measurements in the reaction of MgO and Al_2_O_3_ to form the spinel MgAl_2_O_4_ [122,123]_._

Figure 5c represents the collaboration networks in RFS from countries to institutions and authors. The RFS community is formed by 82 authors and just one of them published single-authored documents (Prof. Chaim from Technion Israel Institute of Technology). On average, each document is co-authored by 5.04 authors with a collaboration index of 3.38 (See Table S5). As shown in the inset of Figure 5c, the documents come from six different countries: The USA, China, Brazil, Spain, India, and Israel. All authors internationally collaborate, besides authors from Israel with two single-country publications [124,125]. More details about author clusters and institutions are shown in Figure 5c. Note that for the sake of visualization, the collaboration network is limited to authors with more than two publications and at least one co-authored document, and unfortunately, Figure 5c does not show the whole RFS networking. From a simple visual inspection, it can be identified that the most prolific authors in number of publications are Prof. Raj and Yoon from the University of Colorado at Boulder, with seven and six publications, respectively (see also Tables S7 and S8). Note that the bubble size is related to the number of publications. Additionally, Figure 5c also unveils the collaboration sub-networks within the RFS community. Analogously to FS, internationalization and collaboration are at the core of this scientific community. For instance, authors from the University of Colorado at Boulder strongly collaborate with Ghose from Brookhaven National Laboratory [117,118,122,123] and authors from Seville University (Spain) [27] and the Federal University of Sergipe (Brazil) [117]. Indeed, the most relevant works of RFS have been a result of these collaboration sub-networks such as the already-mentioned document of *the Journal of Materials Chemistry A* by Gil-Gonzalez et al. [27] or that by Yoon et al. [123], where RFS of MgO and α-Al_2_O_3_ were studied by in situ synchrotron measurements in Brookhaven National Laboratory. This document received 19 global citations, 12 of which are local citations, being one of the most-relevant documents for RFS. Another strong collaboration network is formed by authors from Chinese institutions such as Southwest Jiaotong University, Beijing Institute of Technology, and Northwestern Polytechnical University, who work with An from the University of Central Florida. Their research is primarily dedicated to the preparation of high-entropy oxides by RFS [112,113]. Researchers from Chang’an University working on the preparation of piezoelectric materials also form another cluster [121,126]. It is worth mentioning that China is the second most-prolific country in the number of RFS publications, just behind the USA, at 9 vs. 11 documents. Finally, another important cluster of authors is formed by researchers affiliated to the University of Illinois at Urbana-Champaign, whose works have been devoted to study the transformation of manganese oxides during RFS [127,128].

## 4. Conclusions

FS has constituted a truly breakthrough in materials processing, and since it was proposed for the first time by Prof Raj in 2010, the number of publications has grown exponentially. In this work, we report the scientific landscape of FS by bibliometric analysis, identifying key aspects and peculiarities of the FS community. The target journals where most of the FS papers are published have been pointed out. All of them are dedicated almost exclusively to ceramic materials and are highly ranked journals of the first quartile according to JCR. This highlights the quality of the research carried out in the field. Socially speaking, the knowledge structure has been depicted at different levels, from countries to institutions and authors. A detailed analysis of the interaction of the authors with the highest number of publications is provided. This unveils important collaboration sub-networks that describe the general social structure of the FS scientific community. One of the most striking features is the large number of fruitful national and international collaborations among authors involved in FS with co-authored publications. It seems to be part of the core of this research field. It may be related to the complexity of the flash event that requires the contribution of experts from different disciplines for its understanding and development. The most influential documents in terms of local and global citations have been also identified. A brief description about the topics addressed in those documents is presented as well. Finally, the most recurrent keywords that best describe the document content have been analyzed along with the most-studied materials in 2020 and 2021, identifying the most-tackled and mainstream topics in FS. They reveal that zirconia has always been a hot topic in FS and the documents frequently deal with the understanding of the underlying mechanisms of FS or the non-typical properties granted to flash-sintered materials, such as the abnormal grain growth or defects. As a future research prospect, it is predicted that these topics will be maintained in the next few years. Finally, due to the dramatic scientific growth experienced in RFS in a very short period of time, a bibliometric analysis is provided separately for RFS. A detailed collaboration network is laid out, interestingly showing that the most influential works in the field of RFS are the results of international or national collaboration between authors from different institutions.

All in all, the aim of this work is to draw a general picture of the scientific landscape of FS and RFS by a bibliometric analysis, where the target sources, the most relevant documents, hot and trending topics, and social networking have been identified. To the best of our knowledge, this is the first study of this nature carried out in the field of FS. We believe that this work can be of interest not only for researchers working in the field but also for those who are keen on but not acquainted with FS and RFS. It can be a useful tool to strategize future research and publishing approaches as well as to be quickly updated with this research field, in spite of the high number of scientific publications and the dramatic growth that the field is experiencing.

## Figures and Tables

**Table 1:** Main information.

Description	Results
Period	2010–2021
Documents	318
Sources (Journals, Books, etc.)	63
Average citations per documents	25.07
References	4852
**Authors**	
Authors	670
Author appearances	1498
Authors of single-authored documents	7
Authors of multi-authored documents	663
**Authors Collaboration**	
Single-authored documents	17
Documents per Author	0.475
Authors per Document	2.11
Co-Authors per Documents	4.71
Collaboration Index	2.2

**Table 2:** Authors with the highest number of publications in the analyzed documents set along with their local *h*-index.

Authors	Articles	*h*-Index
Raj R	53	27
Sglavo VM	36	17
Biesuz M	30	14
Jha SK	22	15
Yamamoto T	18	8
Tsakalakos T	16	9
Wang YG	16	9
Wang HY	16	8
Wang H	15	9
Yoshida H	15	9
Charalambous H	14	9
Phuah XL	14	7
Tokunaga T	14	6
Liu JL	13	8
Lebrun JM	12	11
Liu DG	12	8
Chaim R	12	7
Muccillo R	12	7
Grasso S	12	6
Luo J	11	9

**Table 3:** Top 10 Keywords Plus and Authors’ Keywords.

Keywords Plus	Frequency	Author’s Keywords	Frequency
Zirconia/YSZ	160	Zirconia	32
Electrical Conductivity	105	Microstructure	29
Densification	73	Joule Heating	19
Grain-growth	73	Grain Growth	16
Thermal Runaway	71	ZnO	14
Alumina	65	Impedance Spectroscopy	12
Ceramics	49	Ceramics	11
Microstructure	41	Defects	11
ZnO	30	Electrical Conductivity	9
Defect Structure	15	Alumina	8

## Data Availability

Data are contained within the article.

## Supplementary Materials

The following supporting information can be downloaded at: https://www.mdpi.com/article/10.3390/ma15020416/s1, Figure S1: Country scientific production map; Figure S2: Production over time of authors included in Table 2; Figure S3: Historical direct citation network in RFS; Table S1: Most local cited sources; Table S2: Most local cited authors along with their global citations; Table S3: Top-20 most cited documents; Table S4: Most cited documents published in the last two years (2020–2021); Table S5: Word search query for RFS; Table S6: Main information about RFS document sets; Table S7: Authors with three or more publications and local h-index in RFS; Table S8: Most local and global cited authors in RFS; Table S9: Top-5 most cited documents in RFS; Table S10: Top Materials in RFS.
