# Supplementary material for: Flash Sintering Research Perspective: A Bibliometric Analysis
Source: Materials (Basel). 2022 Jan 6;15(2):416. doi: 10.3390/ma15020416 (PMC8779415; doi:10.3390/ma15020416)
Supplement: Supplementary file 1 [file materials-15-00416-s001.zip › materials-1479297-supplementary.pdf]

materials

MDPI

Supporting Information

Flash Sintering Research Perspective: A Bibliometric Analysis

Eva Gil-González [1,2,*], Luis A. Pérez-Maqueda [1,*], Pedro E. Sánchez-Jiménez [1,3,*] and Antonio Perejón [1,3]

1 Instituto de Ciencia de Materiales de Sevilla, Consejo Superior de Investigaciones Científicas−Universidad de Sevilla, Calle Américo Vespucio 49, 41092 Sevilla, Spain. antonio.perejon@icmse.csic.es
2 Departamento de Ingeniería Química, Universidad de Sevilla, Escuela Politécnica Superior, Calle Virgen de África, 7, 41011 Sevilla, Spain
3 Departamento de Química Inorgánica, Facultad de Química, Universidad de Sevilla, 41071 Sevilla, Spain
* Correspondence: eva.gil@icmse.csic.es (E.G.-G.); maqueda@icmse.csic.es (L.A.P.-M.); pedro.enrique@icmse.csic.es (P.E.S.-J.)

Content

1. Figures 2

1.1. Flash Sintering (FS) Figures 2

- Figure S1. Country scientific production map. 2
- Figure S2. Production over time of authors included in Table 2. ...... **Error! Bookmark not defined.**

1.2. Reactive Flash Sintering (RFS) Figures 3

- Figure S3 Historical direct citation network in RFS 3

2. Tables 3

2.1. Flash Sintering (FS) Tables 3

- Table S1. Most local cited sources 3
- Table S2. Most local cited authors along with their global citations. 4
- Table S3. Top-20 most cited documents. 5
- Table S4. Most cited documents during 2020-2021. 7

2.2. Reactive Flash Sintering (RFS) Tables 9

- Table S5. Word search query for RFS 9
- Table S6. Main information about RFS document sets. 9
- Table S7. Authors with three or more publications and local *h*-index in RFS. 9
- Table S8. Most local and global cited authors in RFS. 9
- Table S9. Top-5 most cited documents in RFS. 11
- Table S10. Top Materials in RFS. 12

1. Figures

1.1. Flash Sintering (FS) Figures

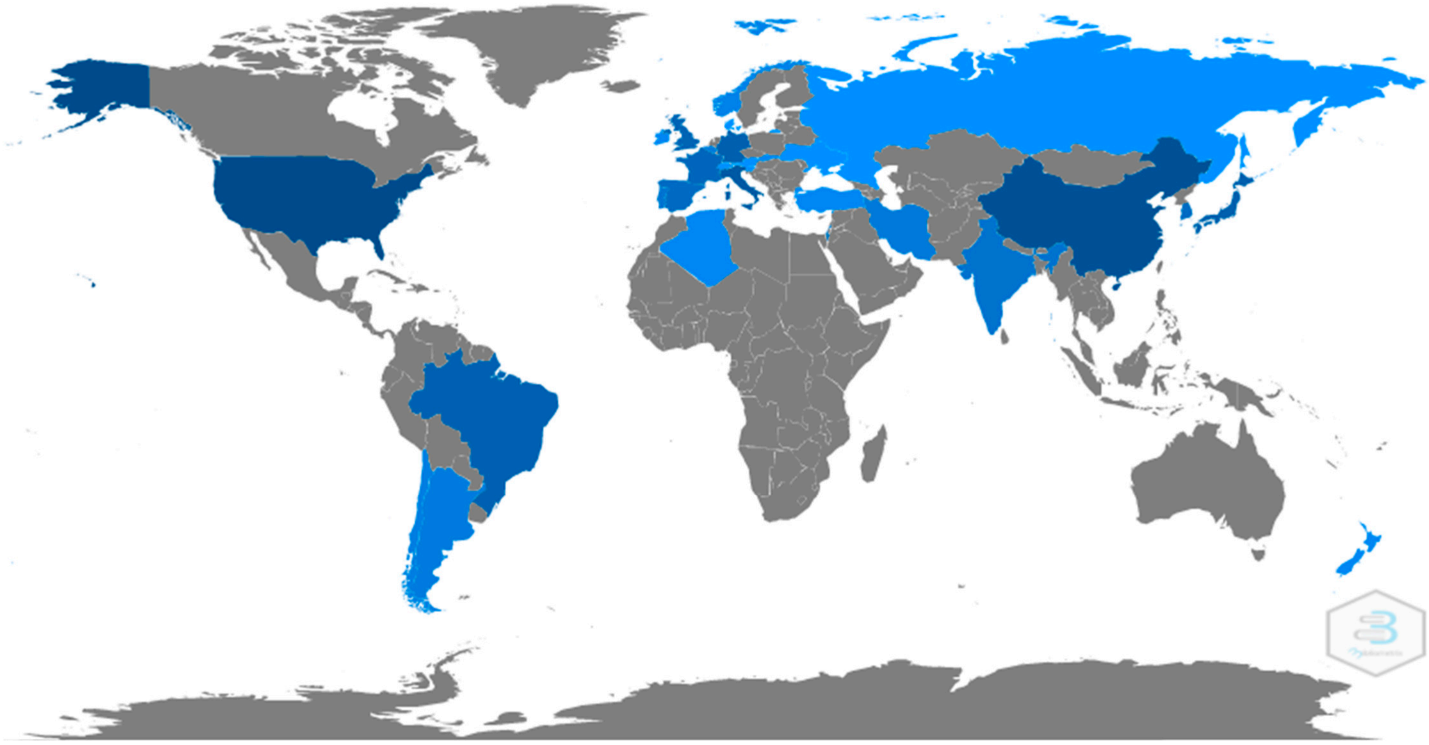

Figure S1. Country scientific production map. The darker, the higher scientific production.

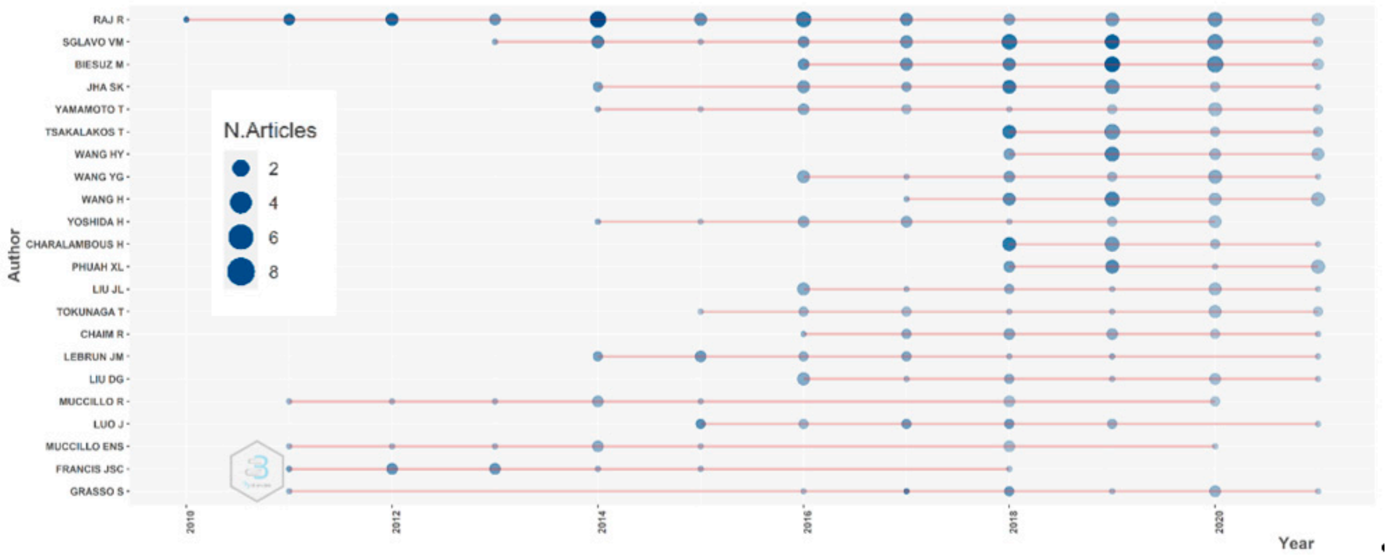

Figure S2. Production over time of authors included in Table 2. The bubble size is proportional to the number of documents, whereas the color to the number of citations (the darker the most cited.

1.2. Reactive Flash Sintering (RFS) Figures

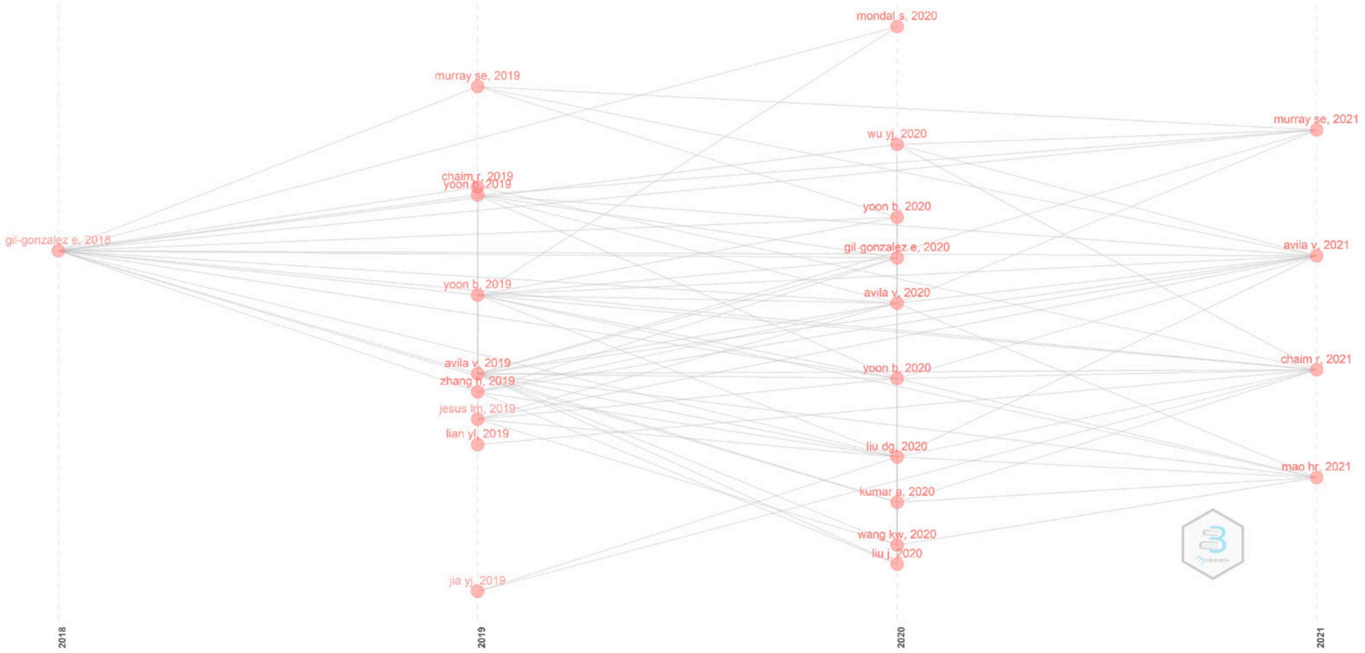

Figure S3. Historical direct citation network in RFS.

2. Tables

2.1. Flash Sintering (FS) Tables

Table S1. Most local cited sources.

Cited Sources	Number of local citation
J AM CERAM SOC	2831
J EUR CERAM SOC	2286
SCRIPTA MATER	793
ACTA MATER	462
CERAM INT	357
J POWER SOURCES	281
J MATER SCI	270
SOLID STATE IONICS	229
J APPL PHYS	221
J CERAM SOC JPN	189
MATERIALS	163
ADV APPL CERAM	147
APPL PHYS LETT	141
MAT SCI ENG A-STRUCT	120
J ALLOY COMPD	116
PHYS REV B	108
SCI REP-UK	99
J ELECTROCHEM SOC	86
J NUCL MATER	70
NATURE	64

Table S2. Most local cited authors along with their global citations.

Cited Author	Local Citations	Global Citations
Raj R.	2123	3012
Cologna M.	812	1236
Sglavo V.M.	757	1069
Francis J.S.C.	604	841
Jha S.K.	439	574
Biesuz M.	420	633
Lebrun J.M.	375	484
Luo J.	332	463
Zhang Y.Y.	296	409
Todd R.I.	282	410
Rashkova B.	270	474
Grasso S.	260	414
Wilshaw P.R.	250	355
Zapata-Solvas E.	250	355
Prette A.L.G.	240	331
Yoshida H.	212	285
Muccillo E.N.S.	204	294
Muccillo R.	204	294
Tsakalakos T.	197	246
Charalambous H.	192	239

Table S3. Top-20 most cited documents.

Authors	Title	Source	DOI	Global Citation (GC)	Local Citation (LC)	LC/GC ratio (%)
Cologna M. et al.	Flash Sintering of Nanograin Zirconia in <5 s at 850°C	J. American Ceramic Society, 2010	10.1111/j.1551-2916.2010.04089.x	474	270	57
Raj R.	Joule heating during flash-sintering	J. European Ceramic Society, 2012	10.1016/j.jeurceramsoc.2012.02.030	241	173	72
Cologna M. et al.	Field assisted and flash sintering of alumina and its relationship to conductivity and MgO-doping	J. European Ceramic Society, 2011	10.1016/j.jeurceramsoc.2011.07.004	230	154	67
Todd R.I. et al.	Electrical characteristics of flash sintering: thermal runaway of Joule heating	J. European Ceramic Society, 2015	10.1016/j.jeurceramsoc.2014.12.022	207	150	72
Yu M. et al.	Review of flash sintering: materials, mechanisms and modelling	Advances in Applied Ceramics, 2017	10.1080/17436753.2016.1251051	178	103	59
Cologna M et al.	Flash-Sintering of Cubic Yttria-Stabilized Zirconia at 750°C for Possible Use in SOFC Manufacturing	J. American Ceramic Society, 2011	10.1111/j.1551-2916.2010.04267.x	166	119	72
Zapata-Solvas E. et al.	Preliminary investigation of flash sintering of SiC	J. European Ceramic Society, 2013	10.1016/j.jeurceramsoc.2013.04.023	148	100	68
Prette A.L.G. et al	Flash-sintering of Co_2MnO_4 spinel for solid oxide fuel cell applications	J Power Sources, 2011	10.1016/j.jpowsour.2010.10.036	141	101	72
Zhang Y.Y. et al.	Thermal runaway, flash sintering and asymmetrical microstructural	Acta Materialia, 2015	10.1016/j.actamat.2015.04.018	138	105	76

	development of ZnO and ZnO–Bi_2O_3 under direct currents					
Francis J.S.C. et al.	Influence of the Field and the Current Limit on Flash Sintering at Isothermal Furnace Temperatures	J. American Ceramic Society, 2013	10.1111/jace.12472	127	100	79
Downs J.A. et al.	Electric Field Assisted Sintering of Cubic Zirconia at 390°C	J. American Ceramic Society, 2013	10.1111/jace.12281	116	88	76
Jha S.K. et al.	The Effect of Electric Field on Sintering and Electrical Conductivity of Titania	J. American Ceramic Society, 2014	10.1111/jace.12682	112	86	77
Narayan J. et. al.	A new mechanism for field-assisted processing and flash sintering of materials	Scripta Materialia, 2013	10.1016/j.scriptamat.2013.02.020	110	72	65
KarakuscU. A. et. al.	Defect Structure of Flash-Sintered Strontium Titanate	J. American Ceramic Society, 2012	10.1111/j.1551-2916.2012.05240.x	107	85	79
Yoshida H. et. al.	Densification behaviour and microstructural development in undoped yttria prepared by flash-sintering	J. European Ceramic Society, 2014	10.1016/j.jeurceramsoc.2013.10.031	104	78	75
Naik K.S. et al.	Flash sintering as a nucleation phenomenon and a model thereof	J. European Ceramic Society, 2014	10.1016/j.jeurceramsoc.2014.04.043	104	85	82
Biesuz M. et al.	Flash sintering of ceramics	J. European Ceramic Society, 2019	10.1016/j.jeurceramsoc.2018.08.048	100	59	59
Hao X.M. et al.	A novel sintering method to obtain fully dense gadolinia doped ceria by applying a direct current	J Power Sources, 2012	10.1016/j.jpowsour.2012.03.006	95	68	72

Schmerbauch C. et al.	Flash Sintering of Nanocrystalline Zinc Oxide and its Influence on Microstructure and Defect Formation	J. American Ceramic Society, 2014	10.1111/jace.12972	94	69	73
Grasso S. et al.	Modeling of the temperature distribution of flash sintered zirconia	J. Ceramic Society Japan, 2011	10.2109/jcersj2.119.144	87	54	62

Table S4. Most cited documents published in the last two years (2020-2021).

**Authors**	**Title**	**Source**	**DOI**	**Global Citation (GC)**	**Local Citation (LC)**	**LC/GC ratio (%)**
Mishra T.P. et al.	Electronic conductivity in gadolinium doped ceria under direct current as a trigger for flash sintering	Scripta Materialia, 2020	10.1016/j.scriptamat.2020.01.007	25	21	84
Lavagnini I.R. et al.	Microstructural evolution of 3YSZ flash-sintered with current ramp control	J. American Ceramic Society, 2020	10.1111/jace.17037	13	16	123
Jongmanns M. et al.	Element-specific displacements in defect-enriched TiO_2: Indication of a flash sintering mechanism	J. American Ceramic Society, 2020	10.1111/jace.16696	17	15	88
Mishra T.P. et al.	Current-rate flash sintering of gadolinium doped ceria: Microstructure and Defect generation	Acta Materialia, 2020	10.1016/j.actamat.2020.02.036	17	15	88
Phuah X.L. et al.	Defects in flash-sintered ceramics and their effects on mechanical properties	MRS Bulletin, 2021	10.1557/s43577-020-00014-y	3	3	100
Xiao W.W. et al.	Ambient flash sintering of reduced graphene	J. Mater. Sci. Technol., 2021	10.1016/j.jmst.2020.04.051	2	3	150

	oxide/zirconia composites: Role of reduced graphene oxide					
Grimley C.A. et al.	A thermal perspective of flash sintering: The effect of AC current ramp rate on microstructure evolution	J. European Ceramic Society, 2021	10.1016/j.jeurceramsoc.2020.11.040	2	3	150
Phuah X.L. et al.	Field-assisted growth of one-dimensional ZnO nanostructures with high defect density	Nanotechnology, 2021	10.1088/1361-6528/abcb2f	1	3	300
Storion A.G. et al.	Influence of the forming method on flash sintering of ZnO ceramics	Ceramic International, 2021	10.1016/j.ceramint.2020.08.210	0	3	0

2.1. Reactive Flash Sintering (RFS) Tables

Table S5. Word search query for RFS.

**TOPIC:** (ELectric Field assisted sintering techniques) *AND* **TOPIC:** (reactive flash sintering) *OR* **TOPIC:** ("reaction flash sintering") *OR* **TOPIC:** ("reactive assisted flash sintering") *OR* **TOPIC:** ("reactive flash sintering") *OR* **TOPIC:** ("reaction assisted flash sintering") *NOT* **TOPIC:** ("Spark Plasma Sintering") **Timespan:** Last 4 years. **Indexes:** SCI-EXPANDED, SSCI, A&HCI, CPCI-S, CPCI-SSH, BKCI-S, BKCI-SSH, ESCI, CCR-EXPANDED, IC.

Table S6. Main information about RFS document sets.

Description	Results
Period	2018-2021
Sources (Journals, Books, etc)	9
Documents	25
Average years from publication	1.28
Average citations per documents	9.08
References	709
**AUTHORS**	
Authors	82
Author Appearances	126
Authors of single-authored documents	1
Authors of multi-authored documents	81
**AUTHORS COLLABORATION**	
Single-authored documents	1
Documents per Author	0.305
Authors per Document	3.28
Co-Authors per Documents	5.04
Collaboration Index	3.38

Table S7. Authors with three or more publications and local *h*-index in RFS.

Authors	Articles	*h*-index
Raj R.	7	6
Yoon B.	6	4
Avila V.	5	3
Jesus L.M.	5	3
Ghose S.	4	3
Liu D.G.	3	3
Liu J.L.	3	3

Table S8. Most local and global cited authors in RFS.

Author	Local Citations	Global Citations
Raj R.	59	124
Yoon B.	31	60
Ghose S.	25	46
Avila V.	23	47
Yadav D.	19	31
Gil-Gonzalez E.	17	47
Perejon A.	17	47

Perez-Maqueda L.A.	17	47
Sanchez-Jimenez P.E.	17	47
Sayagues M.J.	17	46
Jesus L.M.	17	40

Table S9. Top-5 most cited documents in RFS.

Authors	Title	Source	DOI	Global Citation (GC)	Local Citation (LC)	LC/GC ratio (%)
Gil-Gonzalez et al.	Phase-pure $BiFeO_3$ produced by reaction flash-sintering of Bi_2O_3 and Fe_2O_3	J. of Materials Chemistry A, 2018	10.1039/c7ta09239c	46	17	37
Yoon B. et al.	Reactive flash sintering: MgO and α-Al_2O_3 transform and sinter into single-phase polycrystals of $MgAl_2O_4$	J. American Ceramic Society, 2018	10.1111/jace.15974	19	12	63
Lui DG. et al.	Ultrafast synthesis of entropy-stabilized oxide at room temperature	J. European Ceramic Society, 2020	10.1016/j.jeurceramsoc.2020.01.018	19	5	26
Avila V. et al.	Reactive flash sintering of powders of four constituents into a single phase of a complex oxide in a few seconds below 700°C	J. American Ceramic Society, 2019	10.1111/jace.16625	18	11	61
Avila V. et al.	Reactive flash sintering of the complex oxide $Li_{0.5}La_{0.5}TiO_3$ starting from an amorphous precursor powder	Scripta Materialia, 2020	10.1016/j.scriptamat.2019.09.037	15	6	10

Table S10. Top Materials in RFS.

Material	Number of documents
High entropy oxides	6
$BiFeO_3$ related materials	3
Solid Electrolytes	3
$MgAl_2O_4$	3
KNN	2
$Gd_2Zr_2O_7$	2
